# Rational Design of Multicomponent Polymeric Systems Based on a Transient Plasticization Window for Hot-Melt Extrusion

**DOI:** 10.3390/pharmaceutics18060667

**Published:** 2026-05-28

**Authors:** Mark Mandrik, Veronika Makarova, Ludmila Korol, Ivan Krasnyuk, Sergey Antonov

**Affiliations:** 1 A.P. Nelyubin Institute of Pharmacy, Sechenov First Moscow State Medical University, 8-2 Trubetskaya Str., 119991 Moscow, Russia; 2 A.V. Topchiev Institute of Petrochemical Synthesis, Russian Academy of Sciences, Leninsky pr. 29, 119991 Moscow, Russia

**Keywords:** hot-melt extrusion, polymeric premix, low glass transition temperature, melt rheology, polyvinylpyrrolidone (PVP), polyethylene glycol (PEG), hydroxypropylcellulose (HPC)

## Abstract

**Background:** Hot-melt extrusion (HME) is a promising technology for the manufacturing of drug products; however, its application is limited by elevated thermal and shear stresses that may induce degradation of thermolabile active pharmaceutical ingredients. One of the approaches to reducing processing temperatures is the use of polymeric systems with tailored thermal and rheological properties. The aim of the study was to develop an approach for the design of polymeric systems exhibiting a transient plasticization window, enabling a reduction in melt viscosity and improved processability under low-temperature extrusion conditions, followed by the formation of a structurally coherent matrix upon cooling. **Methods:** The compatibility of the initial polymers was assessed using laser microinterferometry. Based on the obtained data, three- and four-component polymeric compositions were designed and prepared by hot-melt extrusion. The resulting materials were characterized by differential scanning calorimetry, melt rheology analysis, and storage stability assessment. Thermal and rheological data were used to iteratively optimize the polymeric systems. **Results:** A four-component polymeric system based on PVP K-29/32, PEG 400, PEG 1500, and HPC EF was developed, suitable for processing by hot-melt extrusion at 70 °C. The final system enabled formation of a homogeneous extrudate, exhibited reproducible rheological behavior, and remained stable in the solid-state during storage, with no evidence of cold flow. **Conclusions:** It was established that, in the design of polymeric systems for hot-melt extrusion, the key factor is not achieving the lowest possible glass transition temperature, but rather the design of a system in which viscosity is transiently reduced under processing conditions and followed by structural stabilization upon cooling. The proposed approach may be applied in the development of polymeric premixes for the preparation of dosage forms by hot-melt extrusion, including those incorporating thermolabile active pharmaceutical ingredients.

## 1. Introduction

Over the past decade, hot-melt extrusion (HME) has become established as a mature pharmaceutical technology capable of producing a wide range of dosage forms, from granules to implants [1]. Growing interest in HME is driven not only by its high productivity and suitability for continuous manufacturing, but also by its ability to modulate the physicochemical properties of active pharmaceutical ingredients (APIs) through the formation of solid dispersions and enhancement of API solubility. This is particularly relevant for poorly soluble compounds belonging to Biopharmaceutics Classification System class II, which account for more than 50% of currently marketed APIs [2]. Moreover, extrusion equipment continues to evolve. Whereas the early transfer of HME from the plastics-processing industry required tens or even hundreds of grams of material and corresponding amounts of API, modern extruders can operate with quantities in the range of hundreds of milligrams. Thus, HME-based drug manufacturing can be readily scaled both up and down, expanding its potential applications in personalized medicine and extemporaneous compounding [3,4], including through the use of API-loaded filaments for 3D printing.

At the same time, one of the major limitations of HME is the requirement for elevated processing temperatures. In most cases, extrusion is performed at temperatures 20–40 °C above the glass transition temperature or melting point of the API carrier composition, typically within the range of 100–200 °C [5]. Such temperatures, combined with shear stresses generated within the extruder barrel, may lead to the thermal and mechanical degradation of heat-sensitive APIs, hereinafter referred to as thermolabile active pharmaceutical ingredients (TAPI) [6]. The literature provides evidence that even a relatively moderate increase in extrusion temperature may induce degradation of certain APIs, thereby restricting the applicability of HME. For example, significant degradation of albendazole during HME at approximately 120 °C has been reported [7]. Gliclazide, an antidiabetic drug classified as a TAPI, also undergoes degradation during extrusion, with the extent of degradation being markedly greater in the amorphous state than in the crystalline form [8]. Another example is glibenclamide, which rapidly degrades upon heating above its melting point during HME [9]. In the case of meloxicam, improved API stability was achieved only by reducing the extrusion temperature to 110 °C [10].

Various strategies have been proposed to reduce extrusion temperature, including the use of volatile solvents or supercritical fluids, which evaporate during processing and temporarily reduce melt viscosity and the effective melting temperature of the TAPI-containing composition [11]. However, such approaches require more complex equipment and efficient solvent removal. Accordingly, the selection and design of thermoplastic carriers with low glass transition or melting temperatures remains the principal strategy for reducing extrusion temperature.

In practice, this is achieved by incorporating low-molecular-weight components with low melting temperatures into the system, that exert a plasticizing effect. The addition of plasticizers reduces melt viscosity and the glass transition temperature of the polymer matrix [12].

There is a growing trend in the field toward the development of carrier systems designed for HME and applicable to a wide range of APIs. A prominent example is Soluplus^®^ (BASF), a graft copolymer of polyethylene glycol, polyvinyl caprolactam, and polyvinyl acetate [13]. In addition, a modified grade of Hypromellose acetate succinate (HPMCAS) with an increased content of hydroxypropoxy groups and an optimized acetyl/succinyl substitution ratio has been patented [14]. This polymer exhibits a lower glass transition temperature compared to conventional HPMCAS, enabling extrusion at reduced temperatures and mitigating TAPI degradation.

The emergence of such ready-to-use matrices allows them to be regarded as intermediates that can significantly streamline and accelerate formulation development. An equally effective approach is the use of premixes—mixtures of polymeric systems and excipients, sometimes also containing APIs. The concept is to pre-form a thermoplastic matrix with predefined properties prior to extrusion, ensuring suitability for HME, including 3D printing applications. Premixes may be prepared using various techniques, such as extrusion, spray drying, granulation, co-crystallization, or co-milling. Moreover, beyond technological advantages, premixes may also offer regulatory advantages. In particular, in the development of generic drug products, premixes can serve as a tool for accelerating market entry. Furthermore, a premix designed as a single multicomponent carrier may be regarded by regulatory authorities as a single excipient with a standardized composition, thereby simplifying the registration process [15].

Despite their evident advantages, current approaches to premix development remain largely empirical and are typically focused on tailoring compositions to specific processing conditions or the properties of individual components [16]. In most cases, the design of such systems is primarily guided by the glass transition or melting temperature [17,18], while the relationship between the phase state of the system, its rheological behavior during heating, and the subsequent structural formation upon cooling remains insufficiently explored [19,20].

Consequently, lowering the extrusion temperature is often achieved at the cost of excessive molecular mobility in the solid state, which may result in instability during storage. These limitations highlight the need for approaches that enable targeted control of rheological properties during processing without compromising structural integrity after cooling.

The aim of the present study was to develop an approach for designing polymeric systems with a transient plasticization window, enabling a reduction in melt viscosity and improved processability under low-temperature extrusion conditions, followed by the formation of a structurally coherent matrix upon cooling.

To implement this approach, the development of a polymeric composition is considered as an independent technological object capable of defining the processability conditions of the system during extrusion. Such an approach can serve as a basis for the subsequent development of dosage forms by HME, including systems containing thermolabile active pharmaceutical ingredients.

Within this study, the phase behavior of binary systems was analyzed using laser microinterferometry, which was employed as a tool for preliminary screening and justification of composition in the design of multicomponent polymeric systems intended for hot-melt extrusion. Based on the obtained data, multicomponent polymeric compositions with tailored thermal and rheological properties were developed and experimentally investigated.

## 2. Materials and Methods

### 2.1. Materials

The following excipients were employed in the development of the polymeric systems: polyvinylpyrrolidone (PVP) K-17 (Plasdone K-17) (USP/EP grade, Ashland Industries Europe GmbH, Schaffhausen, Switzerland), PVP K-29/32 (Plasdone K-29/32) (USP/EP grade, Ashland Industries Europe GmbH, Schaffhausen, Switzerland), polyethylene glycol 400 (PEG 400) (USP/EP grade, Merck, Darmstadt, Germany), polyethylene glycol 1500 (PEG 1500) (USP/EP grade, Clariant AG, Muttenz, Switzerland), hydroxypropylcellulose 80000 (HPC EF) (Klucel EF) (USP/EP grade, Ashland Industries Europe GmbH, Schaffhausen, Switzerland).

### 2.2. Experimental Methods

#### 2.2.1. Laser Microinterferometry

The compatibility of the polymeric excipients was evaluated using laser microinterferometry. The measurement procedure and interferogram analysis were performed according to established methodology [21,22]. Polymer powders (PVP, PEG 1500, and HPC EF) were preheated to 160 °C, 60 °C, and 140 °C, respectively, and compressed to obtain film samples with a thickness of 60–80 µm.

Measurements were carried out during both heating and cooling over a temperature range of 20–250 °C. The moment of contact between the components was considered as the starting point of the diffusion process. A modular laser KLM-A532-15-5 (FTI-Optronic, St. Petersburg, Russia) with a wavelength of 532 nm was used as the light source. The interference patterns were recorded using a digital video camera Levenhuk C800 NG (Levenhuk, Tampa, FL, USA).

#### 2.2.2. Differential Scanning Calorimetry

The thermal properties of the initial components and the resulting polymeric systems were analyzed using differential scanning calorimetry (DSC). Measurements were performed using a Thermal Analysis System TGA/DSC 3+ (Mettler Toledo, Greifensee, Switzerland) over a temperature range of −120 to 200 °C at a heating rate of 10 °C/min under a nitrogen atmosphere with a flow rate of 50 mL/min. Aluminum crucibles with a volume of 40 µL were used, and the sample mass was 10 ± 3 mg.

Samples were analyzed without prior drying in their original state. For each sample, both the first and second heating cycles were recorded, allowing assessment of absorbed moisture content. Cooling between the cycles was carried out according to the standard instrument program. Each test was performed in triplicate, and results are reported as mean ± standard deviation.

#### 2.2.3. Preparation of Polymeric Systems by Hot-Melt Extrusion

Polymeric systems were prepared by hot-melt extrusion using a co-rotating twin-screw extruder Scientific LTE16-36 FAC/00 (Labtech Engineering, Samut Prakan, Thailand) with a screw diameter of 16 mm, a barrel length of 32 L/D, and a die diameter of 2 mm. The extruder was equipped with eight independently controlled heating zones and an additional die heater.

The initial polymers were used without prior drying. Accurately weighed amounts of polymers were manually premixed until visually homogeneous and fed into the extruder hopper. Since the study was of a screening nature and aimed at a comparative evaluation of processability of different compositions, the feed rate was not treated as an independent process parameter and was adjusted during extrusion to maintain stable operation.

**Figure 1:**
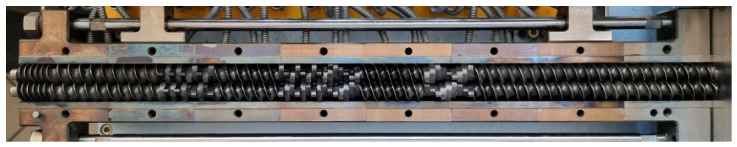
Extruder screw configuration.

The screw configuration was kept constant for all experiments and consisted of four conveying sections alternating with three mixing zones (Figure 1).

The barrel temperature profile was set in the range of 50–120 °C depending on the composition. The extruder was equipped with eight independently controlled barrel heating zones and four melt temperature measurement points, including a sensor located at the die. In addition, the setup was equipped with a melt pressure sensor.

For comparability of processing conditions, extrusion parameters were reported in terms of the maximum set barrel temperature and die temperature, which were considered the upper limit of thermal exposure of the material. This approach allowed the processing conditions to be interpreted as the upper boundary of thermal load, which is critical for the development of systems intended for thermolabile active pharmaceutical ingredients.

After stabilization of the process, defined by reaching steady-state values of pressure and torque (typically within 5 min), the extrudate was collected and cooled to room temperature. The resulting material was pelletized using a strand pelletizer LZ-120 (Labtech Engineering, Samut Prakan, Thailand).

#### 2.2.4. Storage Conditions

The obtained granules were stored in sealed containers at 20 ± 2 °C and 30 ± 2 °C. The temperature of 30 °C was selected as a potentially relevant storage condition.

#### 2.2.5. Rheological Analysis

The temperature dependence of viscosity for all polymeric systems was investigated using a rotational rheometer DHR-2 (TA Instruments, New Castle, DE, USA) equipped with a cone-and-plate geometry (cone diameter 25 mm, cone angle 2°). Rheological measurements were performed during cooling over a temperature range of 100–30 °C at a constant shear rate of 0.1 s^−1^ and a cooling rate of 2 °C/min. The temperature dependence of viscosity was presented in logarithmic coordinates.

Oscillatory rheological measurements were performed for the four-component polymeric systems at 30 °C using the same rotational rheometer equipped with a parallel-plate geometry with a plate diameter of 25 mm. The gap between the plates was set to 0.8 mm. Frequency sweep tests were conducted within the linear viscoelastic region at a strain amplitude of 0.1%, with angular frequency ranging from 0.01 to 100 rad/s. The angular-frequency dependences of the storage modulus (G′), loss modulus (G″), and complex viscosity (η*) were recorded. All rheological measurements were performed in three independent replicates.

#### 2.2.6. Visual Appearance Assessment

The appearance of the granules was evaluated visually against a white background under diffuse lighting. The shape of the granules, surface uniformity, transparency or opalescence, and the presence of visible defects (e.g., cracks, unmelted regions, inclusions) were recorded.

#### 2.2.7. Particle Size Distribution (Sieve Analysis)

The particle size distribution of the granules was determined by sieve analysis in accordance with standard pharmacopoeial methods. A digital electromagnetic sieve shaker, BA200N, and a set of analytical sieves (Cisa Cedaceria Industrial, Barcelona, Spain) with mesh sizes of 3.15, 2.80, 2.50, 2.24, 2.00, 1.80, and 1.60 mm were used.

#### 2.2.8. Loss on Drying

Loss on drying was determined using a moisture analyzer AVG-60 (Gosmetr, Saint Petersburg, Russia). A sample of ground granules (1.5 ± 0.01 g) was dried at 105 ± 1 °C to constant weight. The loss on drying value was calculated as the relative mass difference before and after drying.

#### 2.2.9. Disintegration in Aqueous Medium

The disintegration of the granules was evaluated in accordance with standard pharmacopoeial methods using a disintegration tester ERWEKA ZT 120 (ERWEKA GmbH, Hessen, Germany). Samples were placed in the mesh basket and immersed in deionized water maintained at 37 ± 0.5 °C. The disintegration time was defined as the time required for complete loss of structural integrity of the granules.

## 3. Results and Discussion

### 3.1. Thermal Analysis of Initial Components

**Figure 2:**
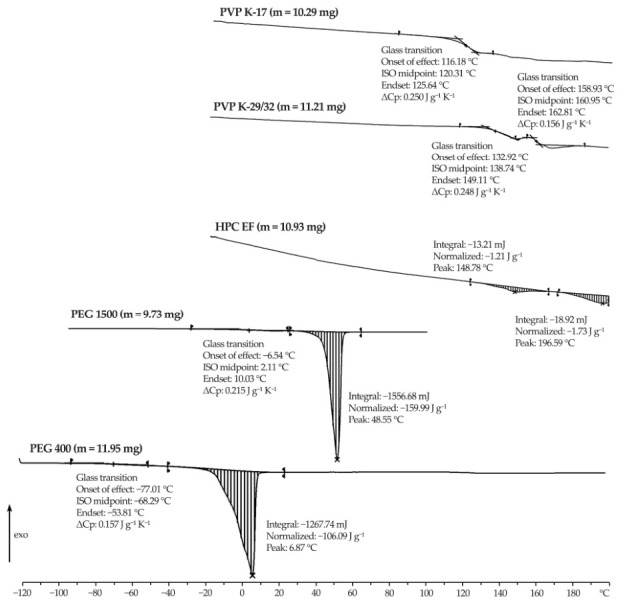
DSC thermograms of the initial components: PVP K-17, PVP K-29/32, HPC EF, PEG 1500, and PEG 400.

Water-soluble polymers widely used in pharmaceutical formulations for immediate drug release were selected for the development of the polymeric systems. The set of initial excipients included polyethylene glycols, polyvinylpyrrolidones, and hydroxypropylcellulose, representing different types of matrices and plasticizing agents. Their thermal properties were evaluated by DSC (Figure 2).

Polyethylene glycols exhibited the expected thermal transitions: PEG 400 was characterized by a low glass transition temperature (–68 ± 0.9 °C) and a melting point at 7 ± 0.9 °C, whereas PEG 1500 showed a glass transition temperature of 2 ± 1.1 °C and a pronounced endothermic melting peak at 49 ± 0.7 °C. These differences reflect the effect of molecular weight on thermal properties and determine the varying efficiency of PEGs of different molecular weights as plasticizers.

For pre-dried PVP K-17, the glass transition temperature was approximately 120 ± 0.8 °C, while for pre-dried PVP K-29/32 it was 139 ± 1.0 °C. In addition, PVP K-29/32 exhibited an additional transition around 161 ± 1.1 °C, which is consistent with molecular weight distribution and heterogeneity of chain mobility.

Hydroxypropylcellulose EF exhibited two characteristic transitions typical of a liquid crystalline polymer: melting of the crystalline phase (149 ± 1.5 °C) and melting of the mesomorphic phase (197 ± 1.6 °C). These observations are consistent with literature data [23,24] and reflect the specific thermodynamic behavior of liquid crystalline polymers.

The obtained thermal parameters were used to define the temperature regimes for extrusion processing and served as input data for the design of multicomponent polymeric systems.

### 3.2. Compatibility Analysis of Initial Components by Laser Microinterferometry

#### 3.2.1. PVP K-17–PEG 400 System

**Figure 3:**
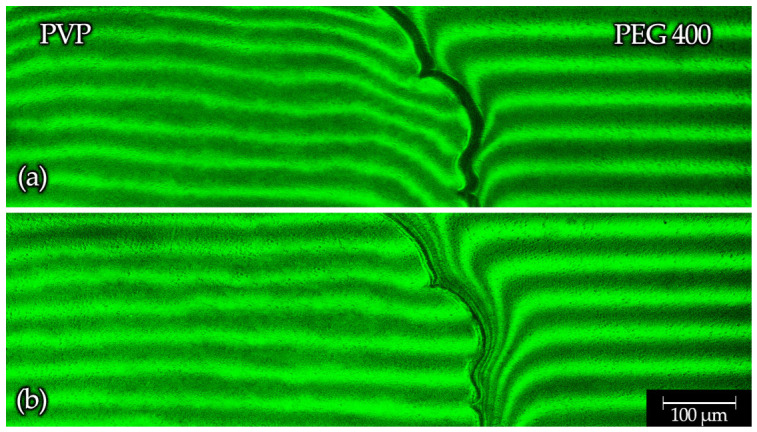
Interferograms of the PVP K-17–PEG 400 system at 45 °C (**a**) and 105 °C (**b**).

The interferograms of the PVP K-17–PEG 400 system are shown in Figure 3. At low temperatures, slight bending of the interference fringes is observed on the PEG side near the phase boundary, indicating the diffusion of PVP into PEG (Figure 3a). With time and increasing temperature, the bending of the fringes becomes more pronounced, while the size of the PVP sample decreases, indicating complete dissolution of PVP in PEG (Figure 3b). A similar behavior is observed for higher-molecular-weight PVP (K-29/32). Thus, PVP is fully soluble in low-molecular-weight PEG already at room temperature, and increasing temperature accelerates this process.

#### 3.2.2. PVP K-17–PEG 1500 System

**Figure 4:**
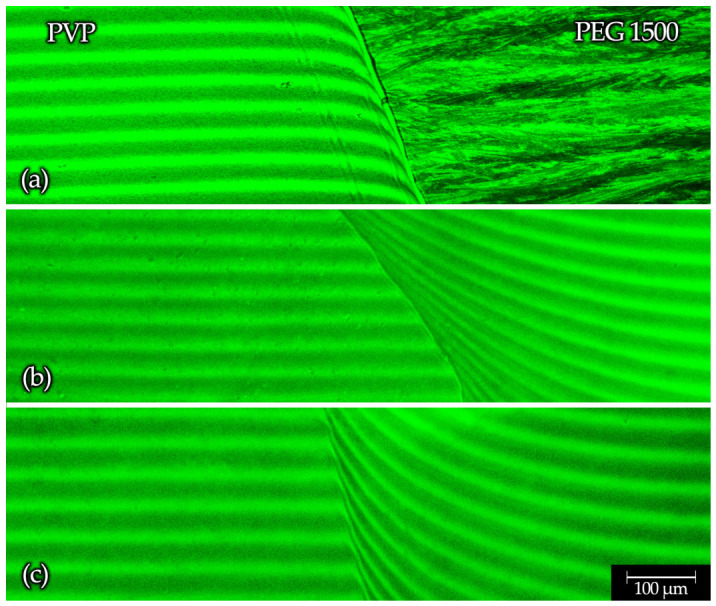
Interferograms of the PVP K-17–PEG 1500 system at 30 °C (**a**), 140 °C (**b**), and 170 °C (**c**).

A series of interferograms obtained for the PVP K-17–PEG 1500 system is shown in Figure 4. At temperatures below the melting point of PEG, no visible interaction between the components is observed. Slight bending of the interference fringes near the phase boundary on the PVP side is attributed to mechanical stresses introduced during cell preparation (Figure 4a). With increasing temperature, the fringes become more uniform, and the interference pattern becomes similar to that observed for low-molecular-weight PEG (Figure 4b): bending of the fringes appears on the PEG side, and the size of the PVP sample gradually decreases. PVP K-17 dissolves in oligomeric polyethylene glycol at temperatures above the melting point of PEG. Further temperature increase beyond the glass transition temperature of PVP leads to the formation of a continuous diffusion front (Figure 4c), confirming full compatibility of the components. A similar behavior is observed for PVP K-29/32. Thus, polyvinylpyrrolidone is fully compatible with oligomeric polyethylene glycol at temperatures above the melting point of PEG.

Previous studies by M.M. Feldstein and co-authors [25,26] have shown that the solubility of PVP in oligomeric PEG is governed by hydrogen bonding between the carbonyl groups of PVP repeating units and the terminal hydroxyl groups of short polyethylene glycol chains, which is consistent with the results obtained in this study. However, an increase in the molecular weight of PEG leads to a reduction in the number of terminal hydroxyl groups and, consequently, to only partial compatibility of PVP with PEG [27]. The interaction of PVP with higher-molecular-weight PEGs, i.e., polyethylene oxides, was not considered in the present study.

#### 3.2.3. PVP K-17–HPC EF System

**Figure 5:**
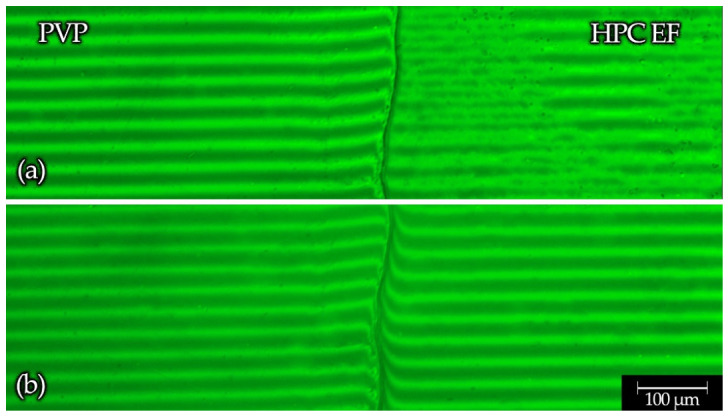
Interferograms of the PVP K-17—HPC EF system at 160 °C (**a**) and 230 °C (**b**).

The interferograms obtained for the PVP–HPC EF system are shown in Figure 5. In the temperature range from room temperature to 205 °C, no significant interaction between PVP and HPC EF is observed, and the interference fringes along the phase boundary remain unchanged (Figure 5a). After the transition of HPC to the isotropic state, in which its liquid-crystalline structure disappears, bending of the fringes on the HPC side becomes apparent, indicating the diffusion of PVP into HPC. With increasing temperature, the extent of fringe bending increases (Figure 5b). According to reference data [28,29], the difference in refractive indices between PVP and HPC is 0.03, which corresponds to the formation of nine interference fringes within the interdiffusion zone. Consequently, in the temperature range of 200–230 °C, the solubility of PVP in HPC EF is estimated to be in the range of 4.4–12.2 ± 0.1 vol.% based on the concentration profile across the diffusion zone. It is known that the transition of HPC to the isotropic state is often accompanied by the onset of thermal degradation [30,31]. In addition, significant degradation of PVP begins at temperatures approaching 250 °C [32,33]. Therefore, further increase in the experimental temperature was not considered meaningful. Thus, above 205 °C only limited dissolution of PVP in HPC is observed, whereas below this temperature the components remain completely incompatible.

#### 3.2.4. HPC EF–PEG 400 and HPC EF–PEG 1500 Systems

**Figure 6:**
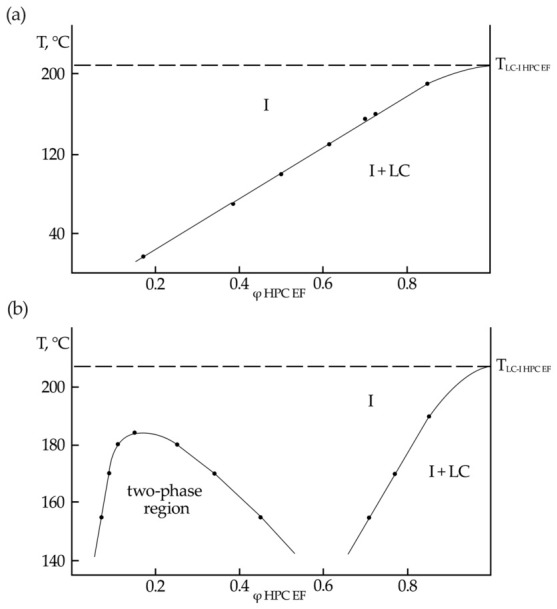
Phase diagrams of HPC EF-PEG 400 (**a**) and HPC EF-PEG 1500 (**b**) systems. Based on the previously reported concept [34].

The interaction of hydroxypropylcellulose with polyethylene glycol 400 and 1500 has been previously investigated using the interferometric micromethod [34]. It was shown that HPC EF is completely soluble in PEG 400. For the HPC EF–PEG 400 system, a liquid crystalline (LC) transition is observed over the temperature range of 20–210 °C. Accordingly, depending on temperature and HPC EF concentration, the system may exist in crystalline, mesomorphic, or isotropic phases. At room temperature, the LC phase begins to form at HPC concentrations above 25% (Figure 6a).

The phase behavior of the HPC EF–PEG 1500 system differs significantly from that of the PEG 400 system (Figure 6b). Increasing the molecular weight of PEG leads to the superposition of two types of phase equilibrium: an amorphous phase (at low and moderate HPC EF concentrations) and a liquid crystalline phase (at higher HPC EF concentrations). The two-phase region narrows with increasing temperature and is characterized by an upper critical solution temperature of 185 °C. Thus, PEG 1500 acts as a weaker solvent for HPC EF compared to PEG 400, and dissolution of HPC EF in PEG 1500 occurs only at temperatures above 100 °C. One possible explanation for this behavior is the intramolecular association of polyethylene glycol chains, as discussed by V.V. Makarova [35] and supported by IR spectroscopy data. Increasing the molecular weight of PEG enhances this effect and reduces the likelihood of interactions between terminal hydroxyl groups and the functional groups of HPC EF.

#### 3.2.5. Summary of Key Findings and Implications for the Design of Multicomponent Polymeric Systems

The microinterferometric investigation of binary systems made it possible to identify the fundamental features of the phase behavior of the initial polymers and thereby establish the framework for the design of multicomponent polymeric systems.

It was shown that polyvinylpyrrolidones exhibit full compatibility with polyethylene glycols (PEG 400 and PEG 1500) at temperatures above the melting point of the latter, which is attributed to the formation of hydrogen bonds between the carbonyl groups of PVP and the hydroxyl groups of PEG. Under these conditions, PEG acts as an effective solvent, enabling the formation of a homogeneous amorphous phase and a reduction in system viscosity.

At the same time, it was established that in PVP–PEG systems containing PEG 1500, compatibility is temperature-dependent. At temperatures below 50 °C, the solvent capacity of PEG 1500 is significantly reduced due to its crystallization.

The PVP–HPC combination was found to be incompatible within the technologically relevant temperature range: mixing was observed only at temperatures close to the isotropic transition of HPC, where the risk of thermal degradation increases. This precludes the formation of a homogeneous matrix in the absence of a compatibilizer.

Hydroxypropylcellulose exhibits full compatibility with PEG 400 and limited compatibility with PEG 1500, particularly at temperatures below 140 °C (Figure 6).

Overall, these results suggest that in multicomponent systems containing PVP and HPC, PEG 400 can act as a compatibilizer, whereas PEG 1500 may perform a dual function: at elevated temperatures, it contributes to viscosity reduction through its solvent action, while upon cooling, it limits system mobility due to partial crystallization.

From a design perspective, this indicates the possibility of forming systems with pronounced temperature-dependent phase behavior, in which changes in the physical state of the components are accompanied by shifts in their solvent capacity and morphological rearrangements.

These findings formed the basis for the development of multicomponent compositions in which the component ratios were selected to enable the formation of a transient plasticization window upon heating while maintaining structural integrity after cooling.

### 3.3. Preparation of Polymeric Systems by Hot-Melt Extrusion

#### 3.3.1. General Approach to Composition Design

The development of the polymeric systems was based on the analysis of the structural and thermal properties of the initial polymers, as well as their phase behavior. The primary objective was the formation of amorphous polymer matrices suitable for the subsequent extrusion-based manufacturing of drug products containing TAPI at temperatures below 80 °C.

To achieve this, three- and four-component systems were systematically developed, differing in the type and molecular weight of polyvinylpyrrolidone, the content of hydroxypropylcellulose, and the ratio of low- and high-molecular-weight polyethylene glycols. The ratio of polyethylene glycols with different molecular weights was considered as one of the factors influencing the rheological properties of the melt and the phase behavior of the system. The results of differential scanning calorimetry and rheological analysis of previously obtained systems were used to iteratively adjust the composition of subsequent systems. Component selection and their quantitative ratios were aimed at achieving a homogeneous matrix with defined melt rheological properties and a stable solid-state behavior during storage. The compositions of the developed polymeric systems are presented in Table 1.

The preparation of polymeric systems by hot-melt extrusion was initially carried out at the lowest possible temperatures, allowing direct assessment of the potential of the resulting matrices for application in low-temperature manufacturing of drug products. An increase in extrusion temperature was considered solely as a means of achieving system homogeneity and was applied only in cases where the formation of a homogeneous melt was not possible under milder thermal conditions. In this context, temperature can be considered not only as a technological parameter, but also as a factor determining the phase state and mutual solubility of the components.

In a number of experiments, the maximum recorded melt temperature slightly exceeded the set barrel temperature (by 4–7 °C), which can be attributed to localized shear heating and differences in temperature measurement accuracy between the heating zones and the melt [36]. These values correspond to peak rather than average process temperatures. The extrusion parameters and the observed melt behavior for all developed systems are summarized in Table 2.

#### 3.3.2. Preparation of Three-Component Polymeric Systems by Hot-Melt Extrusion

Three-component systems were considered as model polymer matrices to evaluate the effect of the type and molecular weight of polyvinylpyrrolidone on the ability to obtain homogeneous systems at reduced temperatures. In these systems, polyethylene glycols (PEG 400 and PEG 1500) acted as plasticizing components governing the melt viscosity, while PVP provided the structural backbone of the amorphous matrix.

System A, containing lower-molecular-weight PVP, enabled the formation of a homogeneous extrudate at temperatures below 70 °C. The values of torque and melt pressure were within the range characteristic of stable material flow and did not indicate extruder overloading. However, during storage at 30 °C, this system exhibited pronounced cold flow and a tendency of granules to agglomerate, indicating insufficient solid-state stability and limiting its practical applicability.

To improve solid-state stability, System B was developed using higher-molecular-weight PVP. This modification resulted in the formation of stable granules that did not exhibit cold flow during storage at 30 °C. However, obtaining a homogeneous extrudate required higher processing temperatures compared to System A and was accompanied by increased torque, reflecting higher melt viscosity due to the increased molecular weight of the structure-forming component [37].

Thus, the three-component systems demonstrated the necessity of balancing low-temperature processability and solid-state stability of the resulting matrix. These findings justified further compositional optimization through the introduction of an additional structural component, HPC EF, which may also contribute to viscosity reduction due to its liquid crystalline behavior.

#### 3.3.3. Preparation of Four-Component Polymeric Systems by Hot-Melt Extrusion

The incorporation of HPC EF and adjustment of the polyethylene glycol ratio in the development of four-component systems was considered as an approach to improve the mechanical stability of the solid state and reduce the tendency toward cold flow and agglomeration, while preserving the possibility of subsequent manufacturing of drug products at reduced temperatures.

System I was used as the initial four-component system. An attempt to prepare this composition at 70 °C did not result in the formation of a homogeneous melt and was accompanied by a substantial increase in torque, reaching approximately 85% of the maximum value. These parameters indicated that the process had exceeded the technologically acceptable operating range. Therefore, System I was prepared at 120 °C, which ensured the formation of a homogeneous extrudate under stable torque and melt pressure conditions.

The obtained result was used as a reference point for the further development of four-component systems. Subsequent four-component compositions were prepared at 120 °C, which ensured the formation of a homogeneous structure and allowed for a consistent comparison of the influence of composition on system properties.

As part of further optimization, Systems II and III were developed by adjusting the quantitative ratios of the components toward a higher total PEG content and modified ratios between PEG grades. This compositional modification affected both the rheological behavior of the melt during extrusion and the solid-state properties of the resulting granules.

For System II, preparation at 120 °C was associated with a lower torque compared with System I at the same screw speed and melt pressure. However, the resulting granules showed a tendency to agglomerate during storage at 30 °C, indicating insufficient stabilization of the solid state at this component ratio.

In System III, the content of PEG 1500 was increased while maintaining the PEG 400 content relative to System II. The extrusion parameters of Systems II and III remained comparable in terms of screw speed, torque, and melt pressure. At the same time, a lower maximum melt temperature was recorded for System III.

The reduction in maximum melt temperature at comparable mechanical load may be attributed to the higher fraction of PEG 1500, which acts as an internal lubricant and contributes to reduced shear heating of the melt during extrusion [19,36,38].

For all four-component systems (Systems I–III), subsequent extrusion of granules was carried out at 70 °C. In contrast to the initial powder mixtures, the preformed compositions could be processed at this temperature to yield a homogeneous extrudate. At the same time, differences between the systems were retained. System I was characterized by an increased torque. System II exhibited more favorable processing parameters; however, the granules showed a tendency to agglomerate during storage. System III demonstrated the ability to be processed at 70 °C while maintaining solid-state stability during storage.

### 3.4. Differential Scanning Calorimetry

#### 3.4.1. Three-Component Polymeric Systems

**Figure 7:**
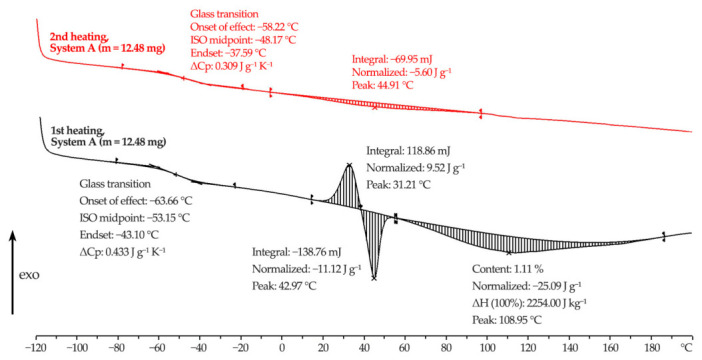
DSC thermogram of System A during the first and second heating cycles.

**Figure 8:**
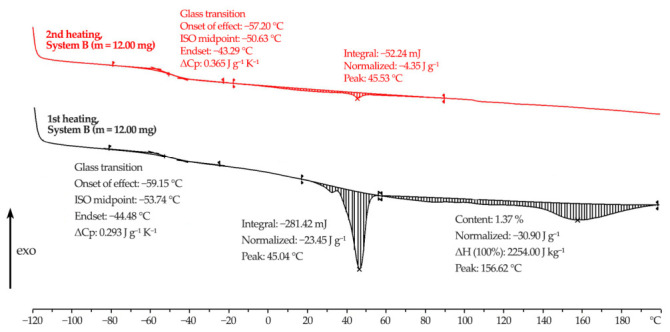
DSC thermogram of System B during the first and second heating cycles.

For Systems A and B, both the first and second heating cycles exhibit a glass transition in the range of −53 ± 0.9 to −48 ± 1.2 °C, indicating a strong plasticizing effect of the PEG components and high segmental mobility of the matrix at temperatures well above Tg (Figure 7 and Figure 8) [39].

In the system based on PVP K-17, an exothermic event with a peak at 31 ± 0.3 °C is observed during the first heating cycle, which can be attributed to cold crystallization or additional ordering of the polyethylene glycol phase. This is followed by an endothermic melting peak of PEG 1500 with a maximum at 43 ± 0.4 °C. Such behavior suggests that the crystallinity of PEG 1500 in this system is weakly expressed and develops only partially upon heating as a result of increased molecular mobility. In contrast, the system based on PVP K-29/32 is characterized by a more pronounced endothermic melting peak of PEG, with a maximum at approximately 45 ± 0.3 °C already during the first heating cycle and in the absence of cold crystallization. This indicates the presence of a significant fraction of crystalline PEG 1500 domains.

After the second heating cycle, the enthalpy of PEG 1500 melting decreases significantly. This indicates a reduction in the degree of crystallinity of the polyethylene glycol phase after standardized thermal treatment. However, small endothermic melting peaks remain, suggesting the presence of residual undissolved PEG 1500 (Figure 8).

Taking the melting enthalpy of the initial PEG 1500 during the first heating cycle (194 ± 13 J/g) as a reference value, the calculated fraction of crystalline PEG 1500 is approximately 0.6 ±0.04 wt.% in System A and 1.2 ± 0.08 wt.% in System B.

Additionally, both systems exhibit an endothermic effect associated with moisture removal, with a content of approximately 1.1–1.4 wt.%. Notably, the system based on PVP K-29/32 shows a shift in this effect to higher temperatures compared to the PVP K-17-based system, indicating stronger water binding and reduced diffusion mobility within the matrix containing higher-molecular-weight PVP.

Thus, the obtained results demonstrate that, at identical compositions, the molecular weight of PVP is the key factor governing the thermal behavior of PVP/PEG systems [40]. The use of PVP K-17 leads to the formation of a more dynamic matrix, in which PEG crystallization is only partially realized upon heating, whereas PVP K-29/32 promotes stabilization of PEG-rich domains and a higher degree of crystallinity already in the initial state.

#### 3.4.2. Four-Component Polymeric Systems

**Figure 9:**
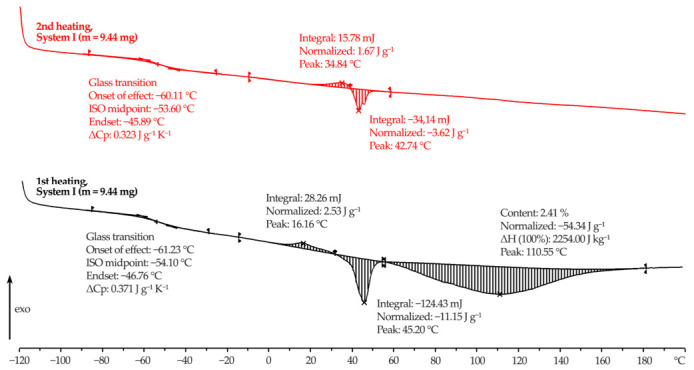
DSC thermogram of System I during the first and second heating cycles.

System I is characterized by the presence of a glass transition at −54 ± 0.7 °C, indicating the formation of a plasticized amorphous matrix (Figure 9). At the same time, moderate endothermic effects are observed in the range of 35–50 °C, corresponding to the melting of the crystalline polyethylene glycol phase, as well as an endothermic effect associated with the removal of 2.4 ± 0.2 wt.% moisture, with a maximum at 110 ± 2.1 °C.

The absence of distinct thermal transitions characteristic of PVP and HPC EF, together with the presence of a single low-temperature glass transition, indicates that both polymers are incorporated into a common amorphous, mechanically stable matrix [41]. This also suggests the absence of separate PVP and HPC EF domains. At the same time, according to extrusion data, the amount of PEG 400 introduced was insufficient to adequately plasticize the matrix and reduce melt resistance to a technologically acceptable level. The fraction of undissolved PEG 1500 in the matrix (0.6 ± 0.04 wt.%) is likewise insufficient to enable processing of System I at an extrusion temperature of 70 °C.

**Figure 10:**
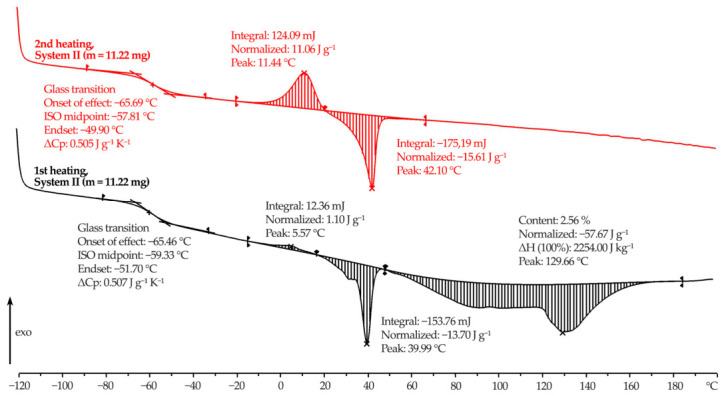
DSC thermogram of System II during the first and second heating cycles.

System II exhibits a glass transition temperature similar to that of System I (Figure 10), but differs in more pronounced thermal effects associated with recrystallization and melting of the polyethylene glycol phase. In the DSC curves, this is manifested by transitions in the range of approximately 30–45 °C, including during the second heating cycle. The presence of these transitions indicates that a fraction of undissolved PEG 1500 (0.8 ± 0.05 wt.%) remains in the matrix, with its melting occurring in a temperature range close to the extrusion processing conditions. In addition, moisture removal is more pronounced in this system and is characterized by a shift in the endothermic peak to approximately 130 ± 3.6 °C, indicating stronger water retention within the polymer matrix and, likely, its involvement in hydrogen bonding with hydrophilic components.

Analysis of extrusion and storage data suggests that an increased content of PEG 400, along with the presence of sorbed moisture, leads to enhanced mobility of polymer chains not only in the melt but also in the solid state [42]. During storage, this manifests as agglomeration of granules at contact points while retaining their overall geometry.

This behavior is consistent with the migration of the plasticizer (PEG 400) to the matrix surface, potentially enhanced by the presence of sorbed moisture, and indicates insufficient stabilization of the composition [43].

**Figure 11:**
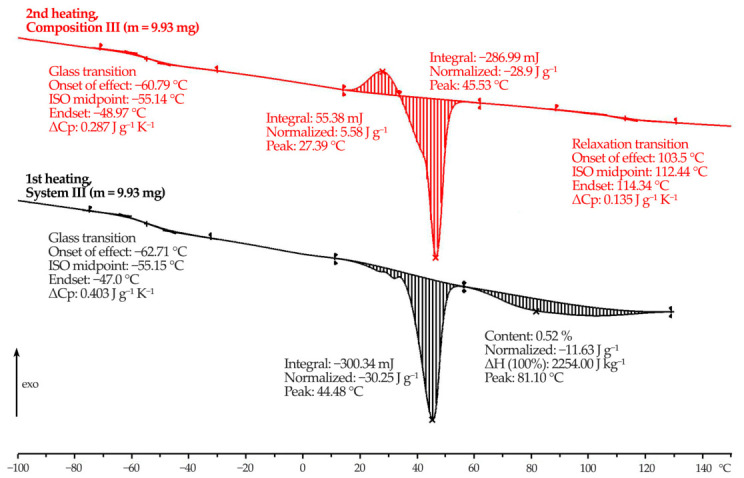
DSC thermogram of System III during the first and second heating cycles.

In the case of System III, DSC thermograms recorded upon heating exhibit a low-temperature glass transition at −55 ± 0.2 °C, indicating the formation of an amorphous polymer matrix with high chain mobility. In addition, during the first heating cycle, endothermic melting effects are observed in the range of 35–55 °C, corresponding to the undissolved crystalline fraction of PEG 1500 (2.9 ± 0.11 wt.%). An additional endothermic effect in the range of approximately 60–130 °C is attributed to the removal of sorbed moisture from the sample, with a content of 0.5 ± 0.08 wt.% (Figure 11).

However, in contrast to Systems I and II, the dehydration effect in System III is less pronounced. This difference is likely related to a distinct organization of components within System III. In this system, a significant fraction of PEG 400 is involved in interactions with PVP and HPC EF, resulting in a reduced number of available hydrophilic sites capable of retaining water.

During the second heating cycle, the low-temperature glass transition remains virtually unchanged, indicating preservation of the structure and composition of the amorphous matrix. In addition, prior to the main endothermic melting peak of PEG 1500 at 46 ± 0.4 °C, a weak exothermic event with a maximum at 27 ± 0.3 °C is observed, which can be attributed to partial structural ordering upon heating. A relaxation transition was observed at 112 °C (0.08 ± 0.055 J·g^−1^·K^−1^), which is likely associated with microphase separation due to the presence of two incompatible polymers combined through a compatibilizer. This transition is weakly expressed, with a maximum recorded thermal effect of 0.135 J·g^−1^·K^−1^. This supports the assumption of overall system homogeneity.

Correlation of DSC data with extrusion parameters indicates that, during processing, molten PEG 1500 contributes to viscosity reduction and stable melt flow, whereas after extrusion and cooling, the system retains sufficient structural rigidity to suppress migration of low-molecular-weight components to the surface. This is consistent with the storage stability data.

Thus, the DSC results demonstrate that differences in extrusion behavior and the tendency toward agglomeration during storage are directly governed by the nature of phase transitions and structural rearrangements occurring upon heating, i.e., by the ability of the system to undergo controlled morphological reorganization. At high HPC content and moderate PEG 1500 fraction (System I), a highly viscous load-bearing matrix is formed, requiring elevated mechanical energy input and leading to shear heating. A slight decrease in HPC content combined with an increase in PEG 1500 (System II) reduces viscosity but increases mobility of the surface layer. Further increase in PEG 1500 content with a concomitant decrease in PVP (System III) results in a regime where PEG ensures efficient processability, while the PVP/HPC-rich phase formed after heating provides a rigid structural framework that suppresses plasticizer migration to the surface.

These findings emphasize that, for the optimization of polymeric systems intended for hot-melt extrusion, the decisive factor is not the absolute value of the glass transition temperature, but the phase state of the system and its evolution during heating.

### 3.5. Rheological Analysis of the Developed Polymeric Systems

#### 3.5.1. Three-Component Polymeric Systems

**Figure 12:**
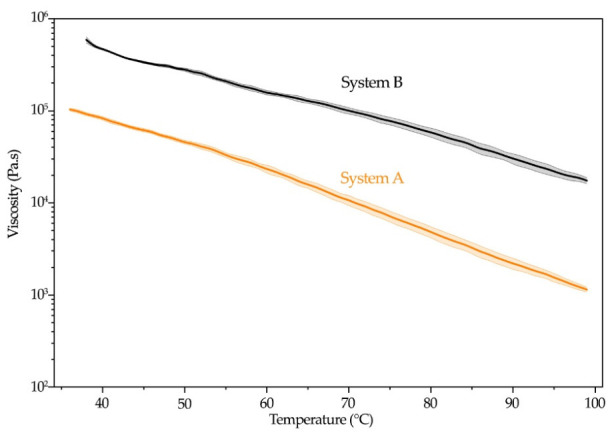
Temperature dependence of melt viscosity for Systems A and B during cooling. Data are presented as mean ± SD (*n* = 3).

The rheological behavior of the three-component Systems A and B differs significantly, despite their similar thermal transitions revealed by DSC (Figure 12).

System A is characterized by lower viscosity across the entire investigated temperature range. Upon cooling from 100 to 30 °C, the increase in viscosity is gradual, and the melt retains high mobility even at temperatures close to storage conditions. Considering the DSC data, which do not reveal additional phase transitions in this range, such behavior is attributed to the formation of a weakly structured amorphous matrix associated with the use of PVP K-17. This rheological profile is consistent with the melt behavior during extrusion and the observed tendency of the material toward cold flow after cooling.

System B exhibits nearly two orders of magnitude higher viscosity at comparable temperatures and a more pronounced increase in flow resistance upon cooling. At the same time, the similar slope of the viscosity curves indicates comparable temperature sensitivity of the melt, while the differences are governed by the baseline viscosity level associated with the higher molecular weight of PVP. The elevated viscosity in the temperature range below 50 °C ensures the preservation of a rigid polymer network and improved solid-state stability, while simultaneously increasing the thermomechanical demands of the extrusion process.

#### 3.5.2. Four-Component Polymeric Systems

**Figure 13:**
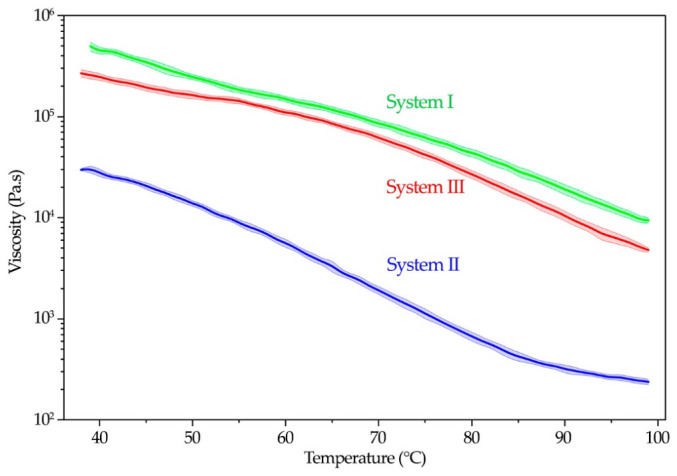
Temperature dependence of melt viscosity for System I, System II and System III during cooling. Data are presented as mean ± SD (*n* = 3).

The temperature dependence of viscosity for all four-component systems exhibits a similar profile: viscosity decreases monotonically with increasing temperature, with no evidence of a change in flow mechanism in the range of 35–100 °C (Figure 13). This suggests a similar nature of the melt across the systems and allows the observed differences to be interpreted as quantitative rather than fundamental.

Across the entire investigated temperature range, System I exhibits the highest viscosity values. At temperatures relevant to extrusion processing, the resistance to flow remains high, which is consistent with the DSC data indicating limited plasticization of the system [20,44]. This rheological behavior confirms the extrusion observations, where System I required elevated thermomechanical input, and processing at reduced temperatures was hindered.

In contrast, System II is characterized by significantly lower viscosity throughout the investigated temperature range. The differences between System II and the other systems persist both in the extrusion temperature region and during subsequent cooling. This behavior indicates substantially higher mobility of the polymer matrix, which ensures high melt flowability but also leads to sustained mobility of components at lower temperatures. This rheological profile is in good agreement with the experimentally observed tendency of System II toward surface stickiness and agglomeration of granules during storage.

System III is positioned close to System I across the entire temperature range, exhibiting only moderately lower viscosity values. At the same time, the shape of the temperature dependence for System III remains similar to that of System I, indicating a comparable structural organization of the melt. Upon cooling, the viscosity of System III increases and remains comparable to that of System I, suggesting the formation of a sufficiently rigid, structurally interconnected matrix with limited component mobility.

Thus, the rheological analysis demonstrates that System III, despite being processable at reduced temperatures, is significantly closer to System I than to System II in terms of viscosity behavior. This indicates retention of a rigid structural organization after cooling, while simultaneously providing technologically acceptable flowability under extrusion conditions.

**Figure 14:**
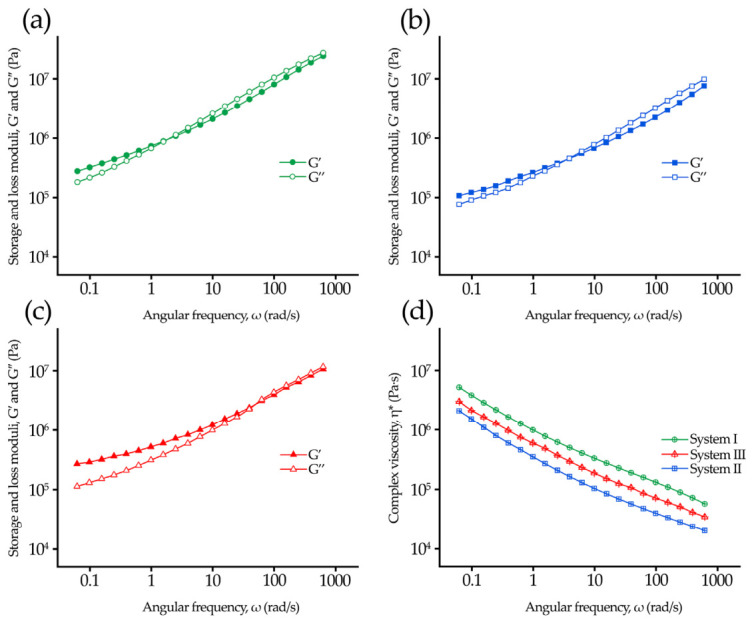
Dynamic rheological behavior of four-component polymeric systems at 30 °C: System I (**a**); System II (**b**); System III (**c**)—storage modulus (G′) and loss modulus (G″) versus angular frequency (ω); complex viscosity (η*) versus angular frequency for Systems I–III (**d**). Data are presented as mean values (*n* = 3).

Dynamic rheological analysis was performed at 30 °C to characterize the viscoelastic behavior of the four-component systems under elevated storage temperature conditions used in this study [19]. The frequency dependences of the storage modulus (G′), loss modulus (G″), and complex viscosity (η*) are presented in Figure 14, while selected quantitative parameters are summarized in Table 3.

For all systems, G′ exceeded G″ in the low-angular-frequency region, indicating the predominance of the elastic contribution and the presence of a structurally interconnected polymer matrix [45]. As angular frequency increased, both moduli increased and the difference between them gradually decreased. In the high-frequency region, G″ approached or exceeded G′, reflecting an increased dissipative contribution under rapid deformation.

Although the overall profile of the frequency dependences was similar, the systems differed in the absolute values of their rheological parameters. System I exhibited the highest complex viscosity, whereas System II showed the lowest values of complex viscosity. System III occupied an intermediate position in terms of complex viscosity.

At the same time, System III demonstrated the largest difference between G′ and G″ in the low-frequency region: at ω = 0.1 rad/s, the difference between G′ and G″ was 155.0 ± 7.0 kPa, compared with 105.4 ± 5.1 and 31.3 ± 1.6 kPa for Systems I and II, respectively. In the high-frequency region, at ω = 100 rad/s, the difference between G″ and G′ was minimal for System III and amounted to 0.410 ± 0.028 MPa, compared with 2.451 ± 0.175 MPa for System I and 1.002 ± 0.084 MPa for System II. These observations indicate that System III maintains a more pronounced elastic response under slow deformations relevant to storage while preserving balanced viscoelastic behavior across the investigated frequency range [45].

**Figure 15:**
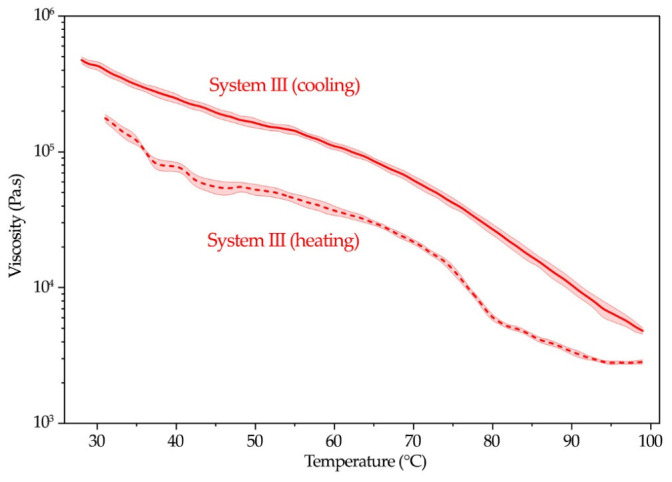
Temperature dependence of melt viscosity for System III during heating and cooling. Data are presented as mean ± SD (*n* = 3).

For further analysis of the rheological behavior and to clarify the nature of the observed temperature dependence of viscosity, System III was additionally investigated under heating conditions (Figure 15).

The presence of slight viscosity hysteresis in System III during heating and cooling is typical of polymeric systems [46]. At the same time, a more pronounced decrease in viscosity is observed upon heating in the 70–80 °C range, emphasizing the role of PEG 1500 as a lubricant.

This feature is consistent with the results of differential scanning calorimetry and laser microinterferometry, according to which melting of undissolved PEG 1500 occurs at around 45 °C, followed by its integration with PVP K-29/32. Thus, the transition of this PEG 1500 fraction into the molten state leads to additional plasticization of the system and a reduction in interchain interactions within the polymer matrix, which manifests as an accelerated decrease in viscosity within this temperature interval.

Taken together, the rheological data, in combination with DSC and extrusion results, support the selection of System III as the composition providing a balanced combination of processability and solid-state stability. Therefore, System III was used in subsequent experimental studies.

### 3.6. Morphological and Functional Characteristics of System III Granules

**Figure 16:**
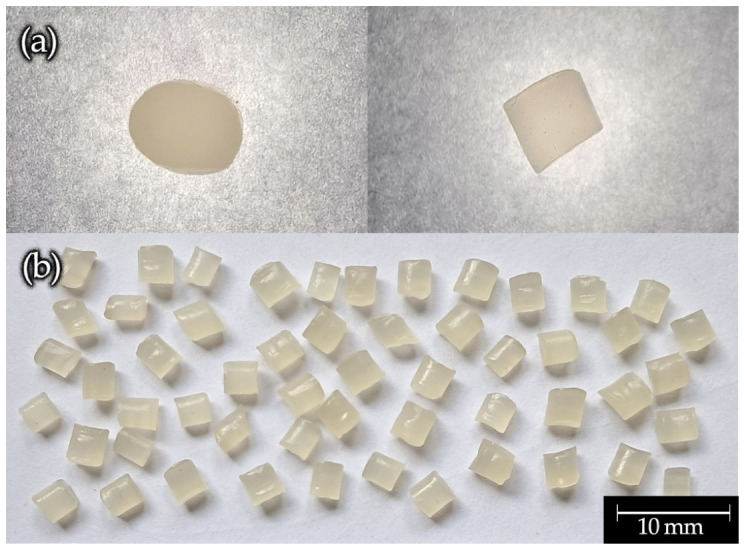
Appearance of System III granules: macroscopic image under transmitted light (**a**), granules arranged in a thin layer on a millimeter-scale background (**b**).

System III granules were uniform in color and appeared as yellow, semi-transparent cylindrical particles with a smooth surface, free of visible defects such as cracks, unmelted regions, or inclusions. The obtained material was characterized by visual uniformity and stable particle shape (Figure 16).

Particle size analysis showed that all granules passed through a 3.15 mm sieve and were retained on a 2.80 mm sieve, indicating a narrow size distribution suitable for subsequent processing steps.

The loss on drying of the final composition was 0.5 ± 0.12%, which is consistent with the thermal analysis data.

Disintegration testing in an aqueous medium at 37 ± 0.5 °C showed that System III granules completely lost their structural integrity within 7 min in all tested samples. These results indicate that the developed composition is suitable for the development of immediate-release drug products.

## 4. Conclusions

In this study, an approach to the design of polymeric premixes intended for low-temperature hot-melt extrusion was proposed and experimentally validated. In contrast to conventional development strategies focused on the properties of APIs, this study was based on designing the polymeric carrier as an independent technological entity with predefined thermal and rheological characteristics. Such an approach allows the polymeric composition to be considered as a platform for the subsequent development of dosage forms by HME.

A key stage of the development was the microinterferometric analysis of binary polymer systems, which enabled direct evaluation of phase compatibility across a wide temperature range. Polyvinylpyrrolidones were found to be fully compatible with low- and medium-molecular-weight polyethylene glycols, whereas direct PVP–HPC combinations remained incompatible under technologically relevant conditions. Microinterferometry further revealed the critical role of PEG as an intermediate component that ensures phase connectivity within the system and defines the allowable compositional space. These findings demonstrate the high predictive value of this method for the preliminary screening of polymer combinations prior to extrusion.

The systematic development and analysis of three- and four-component systems showed that similar glass transition temperatures and the amorphous nature of the matrix are not sufficient criteria for assessing suitability for extrusion.

A central role in the developed systems is played by PEG 1500, which functions as both a compatibilizer and an internal lubricant during extrusion. Its transition through the melting temperature of its crystalline phase provides temporary viscosity reduction and mitigates shear heating during processing. Upon cooling, the retention of a partially crystalline state suppresses the migration of mobile components (PEG 400) and prevents the formation of a continuous low-molecular-weight phase under storage conditions.

Based on the obtained results, the following principle for the design of polymeric premixes intended for low-temperature hot-melt extrusion can be formulated: the polymeric system should provide a temporary reduction in viscosity through thermally activated mobile components under processing conditions, followed by the formation of a more rigid, structurally coherent matrix upon cooling. At the same time, the mobile components should not form a continuous phase or retain high mobility at storage temperatures. This principle is consistent with the observed temperature-dependent phase behavior of the developed systems.

The implementation of this principle in a four-component system based on PVP, PEG 400, PEG 1500, and HPC enabled the development of a polymeric premix that combines processability at 70 °C with solid-state stability during storage. The developed premix can be considered as a basis for the subsequent development of dosage forms by hot-melt extrusion, including cases where reduced processing temperature is a critical technological requirement.

## 5. Limitations and Practical Considerations

The study was conducted without the use of active pharmaceutical ingredients, which allowed the polymeric system to be considered as an independent object and enabled analysis of its phase behavior and rheological properties. The influence of potential interactions between the polymer matrix and APIs was not addressed in this work and requires separate investigation in the context of specific dosage form development.

It should be noted that sorbed moisture affects the mobility of polymer chains and the plasticity of the system, particularly considering the hygroscopic nature of the components used. In practical applications, this factor requires additional control. Specifically, the preparation of compositions should be carried out under controlled low-humidity conditions, while storage should be performed in hermetically sealed packaging. Furthermore, the presence of moisture may affect the stability of APIs susceptible to hydrolysis, which should be taken into account during dosage form development.

The preparation of System III is feasible at 70 °C. However, under such conditions, the physical form of the initial components becomes a critical factor: the use of powdered forms facilitates more rapid homogenization of the system. In contrast, when granulated excipients are used, additional extrusion cycles may be required to achieve a homogeneous structure.

## Figures and Tables

**Table 1:** Compositions of the developed polymeric systems.

System	Components (wt.%)				
	PVP K-17	PVP K-29/32	PEG 400	PEG 1500	HPC EF
A	70	-	20	10	-
B	-	70	20	10	-
I	-	40	20	10	30
II	-	39	22	11	28
III	-	32	22	19	27

**Table 2:** Extrusion parameters of the developed polymeric systems.

System	A	B	I		I (Subsequent Extrusion of Granules)	II	II (Subsequent Extrusion of Granules)	III	III (Subsequent Extrusion of Granules)
Maximum barrel temperature (°C)	65	70	70	120	70	120	70	120	70
Die temperature (°C)	65	65	65	110	70	110	70	110	70
Maximum recorded melt temperature (measured), °C	70	74	73	128	76	127	71	121	72
Screw speed (rpm)	40	40	25	40	25	40	25	40	25
Torque (% of maximum)	27	60	85	15	54	<10	<10	<10	16
Melt pressure (bar)	<10	<10	Up to 40	<10	Up to 30	<10	<10	<10	<10
Observations	Homogeneous extrudate	Homogeneous extrudate	Incomplete homogenization (agglomerates)	Homogeneous extrudate	Homogeneous extrudate	Homogeneous extrudate (low-viscosity melt)	Homogeneous extrudate	Homogeneous extrudate (low-viscosity melt)	Homogeneous extrudate

**Table 3:** Selected rheological parameters of four-component polymeric systems at low and high angular frequencies (30 °C). Values are presented as mean ± SD (*n* = 3).

System	G′−G″ at ω = 0.1 rad/s (kPa)	G″−G′ at ω = 100 rad/s (MPa)	η* at ω = 0.1 rad/s (MPa·s)	η* at ω = 100 rad/s (kPa·s)
I	105.4 ± 5.1	2.451 ± 0.175	3.887 ± 0.034	132.7 ± 6.0
II	31.3 ± 1.6	1.002 ± 0.084	1.542 ± 0.017	40.1 ± 2.4
III	155.0 ± 7.0	0.410 ± 0.028	2.159 ± 0.033	72.5 ± 4.0

## Data Availability

The original contributions presented in this study are included in the article. Further inquiries can be directed to the corresponding author.

## Abbreviations

The following abbreviations are used in this manuscript:

API	Active Pharmaceutical Ingredient
DSC	Differential Scanning Calorimetry
EP	European Pharmacopoeia
HME	Hot-Melt Extrusion
HPC	Hydroxypropylcellulose
HPMCAS	Hypromellose Acetate Succinate
PEG	Polyethylene Glycol
PVP	Polyvinylpyrrolidone
TAPI	Thermolabile Active Pharmaceutical Ingredient
Tg	Glass Transition Temperature
USP	United States Pharmacopeia
